# Generation of Functional Neutrophils from a Mouse Model of X-Linked Chronic Granulomatous Disorder Using Induced Pluripotent Stem Cells

**DOI:** 10.1371/journal.pone.0017565

**Published:** 2011-03-03

**Authors:** Sayandip Mukherjee, Giorgia Santilli, Michael P. Blundell, Susana Navarro, Juan A. Bueren, Adrian J. Thrasher

**Affiliations:** 1 Centre for Immunodeficiency, UCL Institute of Child Health, London, United Kingdom; 2 Hematopoiesis and Gene Therapy Division, Centro de Investigaciones Energéticas, Medioambientales y Tecnológicas (CIEMAT), Madrid, Spain; 3 Great Ormond Street Hospital NHS Trust, London, United Kingdom; University Freiburg, Germany

## Abstract

Murine models of human genetic disorders provide a valuable tool for investigating the scope for application of induced pluripotent stem cells (iPSC). Here we present a proof-of-concept study to demonstrate generation of iPSC from a mouse model of X-linked chronic granulomatous disease (X-CGD), and their successful differentiation into haematopoietic progenitors of the myeloid lineage. We further demonstrate that additive gene transfer using lentiviral vectors encoding *gp91^phox^* is capable of restoring NADPH-oxidase activity in mature neutrophils derived from X-CGD iPSC. In the longer term, correction of iPSC from human patients with CGD has therapeutic potential not only through generation of transplantable haematopoietic stem cells, but also through production of large numbers of autologous functional neutrophils.

## Introduction

The successful application of induced pluripotent stem cell (iPSC) technology in murine models of human diseases, and in generating disease-free autologous cells from patient samples offers considerable potential for development of personalized cell based therapies of monogenic disorders [1]–[9]. In this study, we have studied a mouse model of X-linked chronic granulomatous disorder (X-CGD)[10]. CGD is a group of inherited immunodeficiency disorders resulting from mutations in any one of five subunits of the NADPH-oxidase found in neutrophils and other phagocytic leukocytes. Patients with CGD typically present early in life with recurrent and life-threatening infections due to impaired killing of ingested microbes. Two-thirds of patients with CGD have mutations in the X-linked *CYBB* gene on chromosome Xp21.1 encoding membrane bound *gp91^phox^* (where *^phox^* stands for phagocyte oxidase). X-CGD in human patients can be cured by haematopoietic stem cell transplantation from HLA genotypically-matched donors with high rate of success. Gene therapy using gammaretroviral vectors has also proved to be useful for short term treatment of life-threatening infection, although complicated by insertional mutagenesis. Treatment of those patients without HLA-matched donors remains problematic. In addition to haematopoietic stem cell (HSC) based therapy, refractory infections in CGD patients can be successfully treated using repeated infusions of functional allogeneic neutrophils, although this strategy often results in exaggerated inflammation and allo-immunisation[11]–[14]. In this study, we provide a proof-of-principle that iPSC technology can provide a valuable platform for investigating gene therapeutic approaches in CGD.

## Results and Discussion

**Figure 1:**
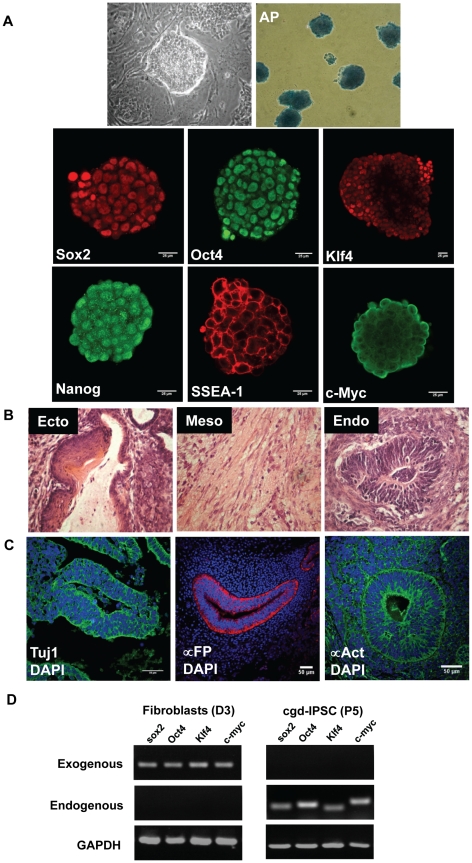
Reprogramming of X-CGD mouse fibroblasts to induced pluripotent stem cells. (A) Images of iPSC showing ES cell like morphology (high nucleus to cytoplasm ratio), high levels of alkaline phosphatase (AP) activity, and expression of pluripotency markers Sox2, Oct4, Klf4, Nanog, SSEA-1, and c-Myc. Bright-field images were acquired with a standard Olympus microscope (20X objective). Fluorescent images were acquired with a Zeiss LSM 710 confocal microscope (25X objective). (B) Haematoxylin & Eosin staining of teratoma sections showing derivatives from three germinal layers (Ecto, ectoderm; Endo, endoderm; Meso, mesoderm). Structures shown include neural tube (ectodermal), striated muscles and cartilaginous structures (mesodermal), and gut-like epithelium (endodermal). (C) Immunofluorescent confocal images of teratoma sections showing antibody staining targeting tissue derivatives present in three germinal layers.Tuj1: neuronal class III β-tubulin; ∝-FP: alpha-fetoprotein; ∝-act: alpha-actinin; DAPI: 4′-6-Diamidino-2-phenylindole.(D) Semi-quantitative reverse transcriptase polymerase chain reaction (RT-PCR) analyses showing silencing of exogenously introduced transgenes as shown by their presence in fibroblasts (three days post transduction, D3), and absence in cgd-IPSC clone (passage five). Specific primers were designed to target regions of retroviral transgenes and endogenous sequences of reprogramming factors. GAPDH, glyceraldehdye 3-phosphate dehydrogenase.

Induced pluripotent stem cells (iPSCs) generated from adult fibroblasts of X-CGD mice were adapted to feeder-free condition for five passages and subsequently characterized for stem cell morphology (round shape, large nucleus, and scant cytoplasm), alkaline phosphatase activity, and expression of pluripotency markers Sox2, Oct4, Klf4, Nanog, SSEA-1, and c-Myc (Fig. 1A). Based on these results, a single clone was selected for future experiments henceforth designated as cgd-iPSC. As control, an iPSC clone from wild-type mice of identical background was also obtained which will be referred to as wt-iPSC. The ability to generate teratoma in immunodeficient mice constitutes an important test of pluripotency. Sub-cutaneous injection of cgd-iPSC into immunodeficient mice generated tumour between the 4^th^ and 5^th^ week and subsequent histological analysis of tumour sections revealed the presence of ectodermal (neural tube), mesodermal (cartilage) and endodermal (gut epithelium) structures as shown in figure 1B. Immunostaining revealed the presence of definitive markers for all three germinal layers in these sections (Figure 1C) thereby confirming the tumour growth as a teratoma. Complete silencing of retroviral transgenes marks the attainment of a fully reprogrammed pluripotent state. This is immensely critical for the employment of iPSC in multi-lineage differentiation protocols[15], [16]. As shown in figure 1D, we could not detect any expression of the exogenous reprogramming factors from the retroviral vectors in cgd-IPSC (passage five) when compared to transduced fibroblasts (day three). Expressions of endogenous reprogramming factor transcripts were consistently detected in cgd-IPSC when cultured and propagated in embryonic stem cell media.

**Figure 2:**
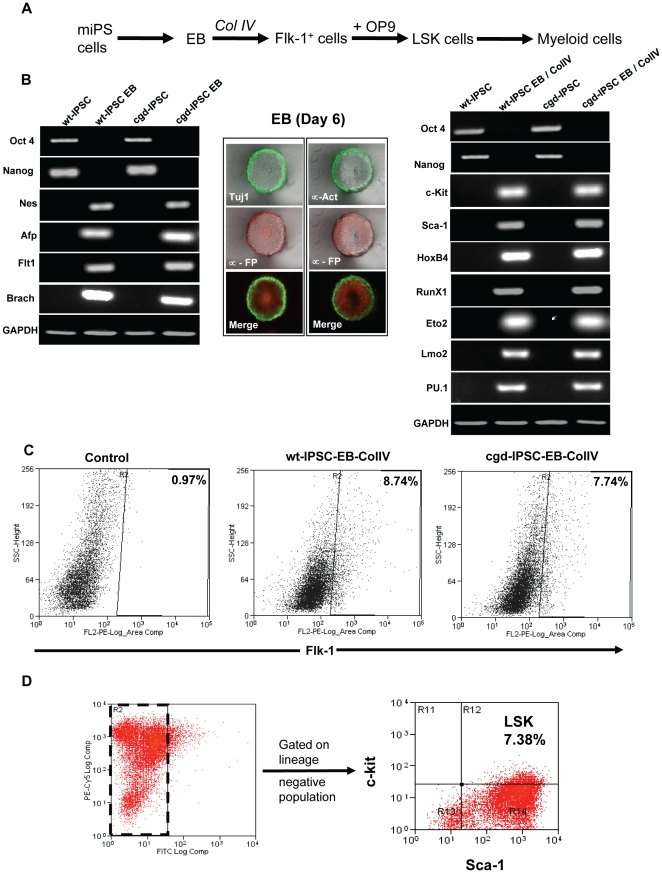
In-vitro differentiation of iPS cells towards mesodermal and haematopoietic lineages. (A) Schematic representation of protocol for haematopoietic differentiation of X-CGD mouse derived iPS cells. EB, embryoid bodies; ColIV, collagen type IV coated plate; Flk-1+ cells, FMS-like tyrosine kinase I positive cells; OP9, M-CSF deficient OP9 stromal cell line; LSK, Lineage negative, Sca-1 positive, c-kit positive cells. (B) Characterization of day six EB cultured under low-attachment conditions. Semi-quantitative RT-PCR analyses showing comparative expression of pluripotency and differentiation markers in wild-type (wt-IPSC) and diseased iPS cells (cgd-IPSC). Left panel shows expression levels of various markers in day six EBs, while those in the right panel shows comparative levels of expression in cells after culturing in collagen IV coated flasks in haematopoietic differentiation medium. Middle panel shows in-situ immunophenotypic staining of six-day EBs with antibodies targeting characteristic antigens expressed in the three germinal layers. (C) FACS analysis showing generation of Flk-1 positive cells from EBs upon collagen IV culture.(D) FACS plot showing derivation of lineage negative, Sca-1 positive and c-kit positive (LSK) population post OP9 co-culture of Flk-1 positive cells.

Our next goal was to differentiate the cgd-iPSC line to cells of myeloid lineage. Myeloid differentiation is a complex process involving coordinated binding of haematopoietic cytokines (notably granulocyte-colony stimulating factor and IL-6) to their cognate receptors in a stage and lineage specific manner resulting in the derivation of mature granulocytes or monocytes/macrophages. The scheme for differentiation is outlined in figure 2A and is adapted from previously published protocols for *in-vitro* haematopoietic differentiation of mouse embryonic stem cells and iPSC with minor modifications[17]. RNA expression profiling of six day old embryoid bodies (EBs) showed down regulation of expression of the pluripotency markers (Nanog and Oct4), with concomitant upregulation in expression of differentiation markers including Nestin (ectoderm), α-fetoprotein (AFP) (endoderm), and mesodermal markers such as Flt-1 (FMS like tyrosine kinase I), and Brachyury (Fig 2B, left panel) confirming the ability of this clone to undergo directed differentiation. *In-situ* immunostaining further confirmed the presence of cellular derivatives from all three germinal layers (Fig 2B, middle panel).

It has been previously shown that reprogrammed murine fibroblasts can be induced to differentiate into cells of the mesodermal lineages when cultured on collagen type IV (ColIV)[18]. We cultured day six EBs on ColIV coated flasks for an additional 4 days. Gene expression profiling at this stage revealed the presence of several haematopoietic markers including c-kit, Sca1, homeobox protein B4 (HoxB4), runt-related transcription factor 1 (RunX1), transcription factor PU.1, LIM domain only 2 (Lmo2) and Eto2 (Figure 2B, right panel). Fluorescence-activated cell sorting (FACS) also revealed the presence of Flk-1 positive progenitor cells. Flk-1 is a candidate marker for mesoderm and hemangioblast cells which are known to have the ability to differentiate into cells of haematopoietic as well as endothelial lineages [19], [20]. The conversion rate (EB to Flk-1 progenitors) was similar in the diseased versus normal iPS derived populations as shown in figure 2C. Sorted Flk-1-positive cells were further co-cultured with the OP9 stromal cell line in defined medium containing haematopoietic cytokines. Co-culture with OP9 has been shown to confer myelo-erythroid potential to mouse haematopoietic stem cells [21], [22]. Between days three and five of cgd-IPSC and OP9 co-culture, we were able to detect the presence of a lineage negative, Sca-1 positive, c-kit positive (LSK) population of nascent haematopoietic stem cells (7.38%) as shown in figure 2D. This compared closely to the numbers (9.9%) obtained from wt-IPSc (data not shown).

**Figure 3:**
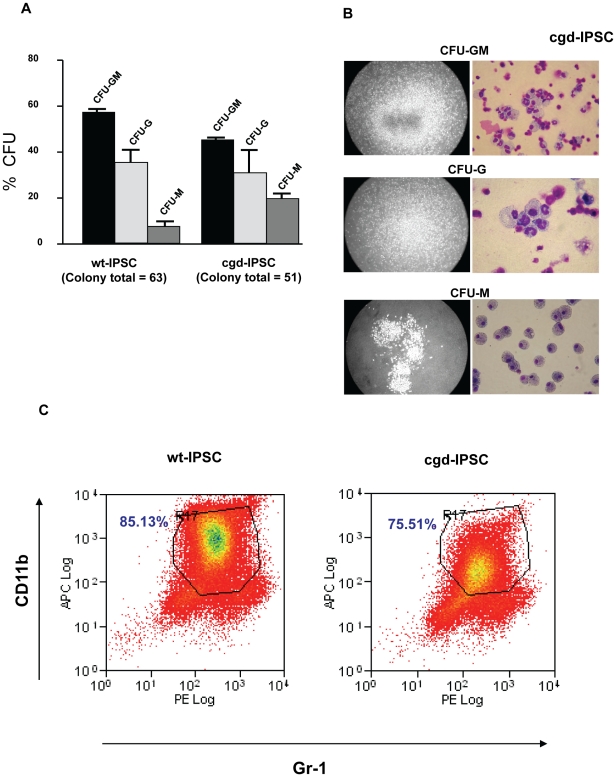
Myeloid differentiation of iPS cell derived LSK cells. Colony Forming Unit (CFU) assay was performed to test the ability of LSK cells to undergo terminal differentiation in semi-solid methylcellulose media containing suitable cytokine cocktail. (A) Graph showing the relative distribution of CFU-GM (granulocyte/macrophage), CFU-G (granulocyte), and CFU-M (macrophage) colonies scored on the basis of their morphology from three independent cultures. Error bars denote standard error of mean (SEM). (B) Representative cytospin preparation of Diff-Quik stained cells isolated from characteristic colonies showing presence of granulocytes and macrophages including and not restricted to mature and functional neutrophils. (C) FACS plot showing presence of double positive Gr-1 (granulocyte differentiation marker) and CD11b (myeloid and NK cells marker) cells isolated from colonies growing in methycellulose differentiation medium.

LSK cells were assayed for their clonogenic haematopoietic potential by culturing in semi-solid medium containing suitable cytokines promoting myeloid differentiation. It has been shown previously that lineage negative, Sca-1 positive bone marrow cells consist of a virtually pure population of multilineage haematopoietic stem cells[23]. Between one to two weeks later, colonies were scored based on their morphology (CFU-GM, CFU-G, and CFU-M). Both wt-iPSC and cgd-iPSC showed similar distribution in the formation of CFUs (Figure 3A). Cytospin preparations from the cgd-iPSC-derived colonies showed the presence of mature neutrophils, macrophages and monocytes (Figure 3B). This was confirmed by flow cytometric analysis which showed the presence of double positive Gr-1 (myeloid differentiation antigen) and CD11b (macrophage and granulocyte marker) cell populations (71%) which compared closely to those derived from wt-iPSCs (85%) (Figure 3C). This demonstrates as expected that the inherent genetic defect does not interfere with the potential of cgd-iPSC to generate terminally differentiated granulocytes/monocytes.

**Figure 4:**
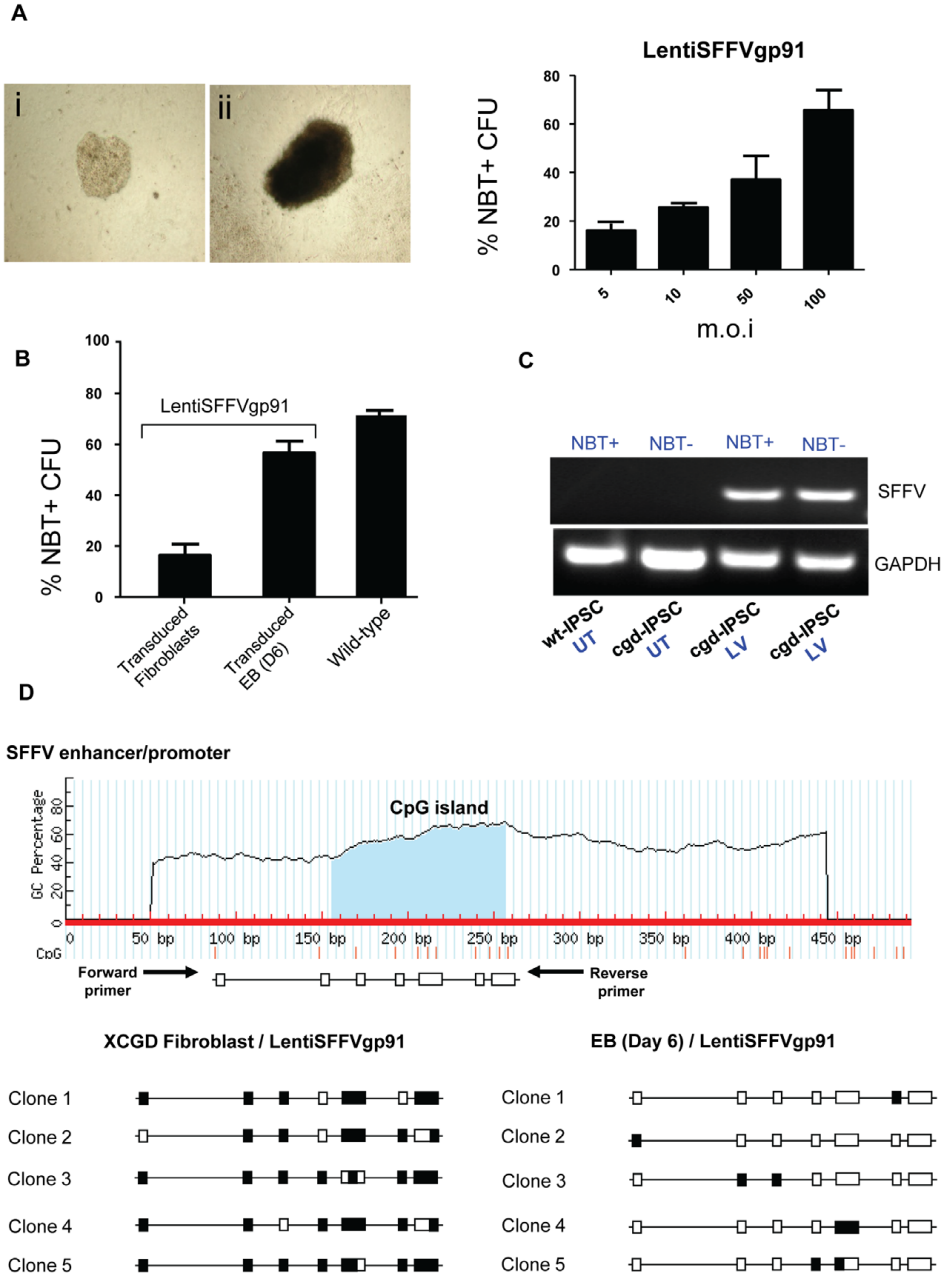
Correction of X-CGD phenotype in-vitro. (A) Phase contrast images showing results of Nitroblue tetrazolium (NBT) assay. Left panel showing untransduced NBT negative (i) and LentiSFFVgp91 transduced NBT positive (ii) colony. Graph showing positive correlation between the multiplicity of infection (m.o.i) and generation of NBT positive colonies. (B) Comparative analysis of the efficiency of disease phenotype correction by lentiviral transduction of pre- and post-reprogramming targets (fibroblasts vs EBs respectively), as assayed by percentage of NBT positive colonies. Restoration of NADPH oxidase activity was compared to number of NBT positive colonies obtained from wild-type iPSC derived myeloid progenitors. Error bars denote standard error of mean (SEM). (C) Agarose gel electrophoresis image showing PCR amplification of SFFV promoter sequence in genomic DNA of NBT negative CFUs confirming the presence of integrated vector. UT, untransduced; LV, Lentivector transduced. (D) Bisulphite genomic sequencing analysis showing higher degree of methylation in lentiviral promoter (SFFV) when vector transduction was performed at the fibroblast stage compared to EB stage. Filled boxes indicate methylated CpG dinucleotide.

**Figure 5:**
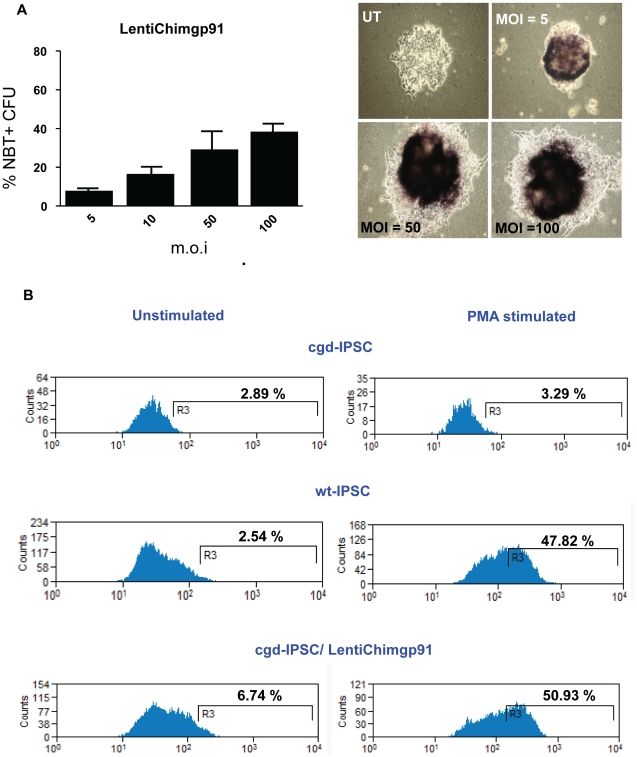
Efficacy of novel lentiviral vector for generation of disease-free haematopoietic cells. Embryoid bodies were transduced with lentiviral vector encoding codon optimized *gp91^phox^* from an internal chimeric promoter (LentiChimgp91). (A) Graph showing positive correlation between the multiplicity of infection (m.o.i) and generation of NBT positive colonies. Error bars denote standard error of mean (SEM). Phase contrast images of CFU-GM colonies showing results from NBT assay. (B) FACS derived histograms showing results of dihydrorhodamine123 (DHR) test upon phorbol myristate acetate (PMA) stimulation. DHR loaded neutrophils upon PMA stimulation show a shift in fluorescence due to reduction of DHR123 by respiratory burst generated by functional neutrophils.

For gene correction, EBs derived from cgd-iPSC (six days) were transduced with a self-inactivating (SIN) lentiviral vector encoding a codon optimized *gp91^phox^* transgene expressed from an internal spleen focus forming virus (SFFV) derived promoter (LentiSFFVgp91) at different multiplicities of infection (m.o.i). CFU derived from transduced EBs were analysed for functional activity of the NADPH-oxidase by Nitroblue Tetrazolium (NBT) assay. CFUs derived from lentivirus transduced EBs showed positive staining in the NBT assay (Figure 4A, ii) compared to untransduced control cells (Figure 4A, i). We also observed a positive correlation between the percentage of NBT positive CFUs and increasing m.o.i (Figure 4A). This is most likely due to better transduction efficiency of EBs. Surprisingly, when compared against percentage of NBT positive CFUs (70.2%±3.8) derived from wild-type iPSC (Figure 4B), we observed three-fold lower efficiency of correction when fibroblasts (18.3%±3.4), rather than EBs (57.5%±9.1) were transduced with the lentivector encoding *gp91^phox^*. In this case we were able to detect the presence of integrated provirus in the majority of NBT-negative CFUs derived from fibroblast-transduced samples as shown in figure 4C (lane NBT-, LV). Analysis of the CpG islands in the SFFV promoter/enhancer region revealed extensive methylation (indicated by filled boxes in figure 4D, left panel) indicating that epigenetic silencing during reprogramming and subsequent differentiation is likely to have occurred[24], [25]. In contrast, presence of integrated vector could not be detected in any of NBT-negative clones derived from transduced EBs, indicating that they were not actually transduced (data not shown). Furthermore, the levels of vector methylation in the NBT positive clones derived from the same EB cultures was much lower (Figure 4D, right panel). We also used a newly described SIN lentivector regulating the expression of *gp91^phox^* from a chimeric promoter consisting of the myeloid-specific minimal c-fes promoter fused to cathepsin-G regulatory sequences[26]. EBs transduced with this vector showed similar levels of correction of disease phenotype as revealed by NBT assay (Figures 5A and 4A). We also estimated the ability of the chimeric vector to restore respiratory burst activity using a more quantitative assay which measures change in fluorescence of dihydrorhodamine 123 (DHR)-loaded CD11b/Gr1 + neutrophils after PMA stimulation. As shown in figure 5B, untransduced cgd-iPSC-derived granulocytes showed no significant difference between PMA stimulated and unstimulated samples (top panel); wt-iPSC-derived cells showed a significant shift in fluorescence upon stimulation indicating reduction of DHR123 to rhodamine by reactive oxygen species(middle panel); DHR-loaded neutrophils derived from cgd-iPSC EBs transduced by the chimeric vector showed a shift in fluorescence peak upon stimulation that was comparable to that of wild type cells (45.28% of total number of cells for wt-iPSC compared to 44.19% of vector transduced cgd-iPSC).

In this study we have demonstrated successful generation of iPSCs from X-CGD mice and their induced differentiation into haematopoietic myeloid progenitors and functional neutrophils. We observed no significant difference between iPSCs generated from wild-type mice and those generated from X-CGD mice regarding their ability to differentiate. We have also demonstrated *in-vitro* restoration of NADPH-oxidase activity in derived X-CGD granulocytes by transduction of the iPSCs (preferentially at EB stage) with lentivectors expressing the *gp91^phox^*, and therefore have provided a proof-of-principle that iPSC technology can be combined with gene therapeutic approaches for modelling a rare primary immunodeficiency such as CGD. Vector design is an important consideration for effective genetic correction of cells prior to reprogramming as epigenetic modifications during this process may lead to irreversible silencing. Utilisation of iPSC for screening alternative vector configurations may therefore prove to be particularly useful. Further investigation is needed to determine if haematopoietic stem cells (HSCs) generated from the gene corrected iPS cells can be employed for long-term multi-lineage reconstitution of murine models and functional correction of the disease phenotype (i.e. susceptibility to invading microorganisms). Derivation of HSCs from human embryonic stem (hES) cell lines and iPSC remains a significant hurdle to translation of this technology, but has obvious clinical application for transplantation. Several groups have recently reported the successful derivation of mature and functional neutrophils from hES cell lines[27], [28], as well as from human iPSCs[29]. Production of large numbers of gene-corrected mature myeloid effector cells from patients with CGD may therefore also have significant therapeutic potential.

## Materials and Methods

### Ethics statement

All animals were handled in strict accordance with good animal practice as defined by UK Home Office Animal Welfare Legislation, and all animal work was approved by the Institutional Research Ethics Committee (Institute of Child Health, University College London, UK) and performed under project license number 70/7024.

### Vectors

Generation and titration of retroviral vectors used for reprogramming adult fibroblasts isolated from X-CGD mice, and the self-inactivating (SIN) lentiviral vectors used for the purpose of correcting the X-CGD genetic defect have been previously described [5], [26]. Briefly, for retroviral vector generation, 293T cells were transiently transfected with packaging plasmid expressing Murine Leukemia virus-gag/pol, Vesicular stomatitis virus derived G protein (VSV-G) expressing plasmid pMD.G2, and pMXS-based reprogramming vectors [5] using standard polyethyelnimine (PEI) protocol. Viral supernatant was harvested forty-eight hours and seventy-two hours post-transfection and concentrated by ultra-centrifugation, and stored in aliquots in -80°C. For VSV-G pseudotyped lentiviral vector generation an identical protocol was followed employing a HIV-1 gag/pol packaging plasmid pCMVΔR8.74, pMD.G2, and transducing vector pCCLChimc.o.gp91^phox^ or pCCLSFFVcogp91^phox^ [26] (referred in the text as LentiChimgp91^phox^ and LentiSFFVgp91^phox^ respectively).

### Animals

X-CGD mice B6.129S6-Cybb^tm1Din/J^ deficient on *gp91^phox^* [9] and wild-type Blck6Ly5.2 mice were obtained from Jackson Laboratories (USA).

### Cell culture and iPSC generation

Adult mouse fibroblasts isolated from X-CGD mice using standard protocol were cultured in Dulbecco's modified Eagle's medium (DMEM) [Invitrogen, CA] supplemented with 10% fetal calf serum (FCS) and 1% penicillin-streptomycin for two to three passages under standard conditions and then frozen into aliquots. The day before transduction, 5×10^4^ fibroblasts were seeded on each well of a six-well plate. Next day, the cells were infected with a cocktail of retroviral reprogramming vectors together with protamine sulphate. After three days, transduced cells were split into gelatine-coated six-well plates and maintained in ESGRO Complete Plus Clonal medium [Millipore] with regular medium changes until colonies appear and are ready for clonal isolation and expansion. The iPSC were cultured feeder-free on gelatine-coated flasks in ESGRO Complete Plus Clonal medium at 37°C, 5% CO_2_. Cells were passaged using Accutase [Millipore].

### Teratoma generation

1×10^6^ iPS cells in matrigel (per site per injection) were injected subcutaneously into immunodeficient mice [common γ-chain^-/-^,RAG2^-/-^, C5^-/-^]. Mice were sacrificed within 4-6 weeks after injection and teratoma was dissected and processed for haematoxylin/eosin staining and immunostaining with suitable antibodies. Following primary antibodies along with suitable secondary conjugated antibodies were used in this study: anti-tubulin beta III isoform (Tuj1) (ectodermal derivatives), anti-alpha-actinin (mesodermal derivatives), anti-alpha fetoprotein (endodermal derivatives) (all from Millipore). As control for non-specific staining, fluorophore-conjugated secondary antibodies were employed in absence of primary antibodies. Teratoma images were processed with ImageJ software.

### In vitro differentiation

For Embryoid Body (EB) formation, iPSC were dissociated using enzyme free cell dissociation buffer, resuspended in LIF free medium and cultured at low attachment condition. For the collagen IV cultures, iPSC derived EBs were triturated and transferred to collagen IV-coated flasks [BD Biosciences]. For OP9 co-culture, Flk1-positive iPSC derived from collagen IV cultures were enriched by fluorescence activated cell sorting (FACS) and plated on OP9 stromal cells in 24 well plates at a concentration of 2 × 10^4^ cells per well in 1 ml of α–minimum essential medium [Invitrogen] supplemented with 20% FBS, 1% penicillin/streptomycin, interleukin (IL)-3 (5 ng/ml), and granulocyte colony-stimulating factor (GCSF; 10 ng/ml) for up to 7 days. Half of the medium with cytokines was replaced every 2 days. OP9 cells were obtained from American Type Cell culture (ATCC).

### FACS analysis

Cells were pelleted by centrifugation, washed in PBS, and stained with fluorescein isothiocyanate (FITC)-, phycoerythrin (PE)-, or allophycocyanin (APC)-conjugated monoclonal rat anti-mouse antibodies against Flk1, Sca1, c-Kit, Gr-1, and CD11b. Nonspecific fluorochrome and isotype matched Ig's served as controls. Dead cells were excluded from analysis and all analysis was performed using a BD LSRII flow cytometer. FACS data were analyzed using FlowJo software.

### Colony forming unit (CFU) assay

To determine the myeloid potential, iPSC derived Flk-1^+^, lineage negative c-kit^+^, Sca-1^+^ cells were plated in methylcellulose medium containing cytokines for myeloid differentiation [Methocult GF M3534; StemCell Technologies]. Colonies were scored between 1-2 weeks.

### NBT assay

Methylcellulose plate containing the CFUs was overlaid with one-fifth volume of NBT-saturated RPMI-1640 medium (Invitrogen) containing 100 ng/ml PMA (Sigma) and 5% human serum albumin (Baxter Healthcare, Deerfield, IL, USA) and incubated at 37°C. After 30 minutes of incubation, the dishes were examined on an inverted microscope, and the colonies with blue formazan precipitates were scored as NBT-positive.

### DHR assay

CFU colonies were extracted from methylcellulose cultures and washed rigorously to get rid of any methylcelluose. The cells were incubated with 30 µM DHR at 37°C for 5 min and stimulated with 5 µg/ml PMA at 37°C for 30 min.

### Bisulphite sequencing

Genomic DNA was isolated from cells using the DNeasy kit (Qiagen) and sodium bisulfite treatment of genomic DNA was performed using the EpiTect bisulfite kit (Qiagen). PCR primers were designed based on converted sequences. Primers were chosen to cover partial length of the SFFV covering the enhancer region (containing 11 CpG sites). PCR conditions were as follows: first round, annealing temperature 58°C, 33 cycles; second round, annealing temperature 56°C, 32 cycles. The PCR primer sequences used were as follows: (i) SFFV promoter: Forward, 5′GGG GGA ATG AAA GAT TTT ATT TG 3′; Reverse, 5′ TCT AAA AAC CAT CTA CTC TTA ACC T 3′. PCR products were cloned into TOPO-TA vector (Invitrogen) and sequenced.

## References

[1] Hanna J, Wernig M, Markoulaki S, Sun CW, Meissner A (2007). Treatment of sickle cell anemia mouse model with iPS cells generated from autologous skin.. Science.

[2] Staerk J, Dawlaty MM, Gao Q, Maetzel D, Hanna J (2010). Reprogramming of human peripheral blood cells to induced pluripotent stem cells.. Cell Stem Cell.

[3] Aasen T, Belmonte JC (2010). Isolation and cultivation of human keratinocytes from skin or plucked hair for the generation of induced pluripotent stem cells.. Nat Protoc.

[4] Takahashi K, Tanabe K, Ohnuki M, Narita M, Ichisaka T (2007). Induction of pluripotent stem cells from adult human fibroblasts by defined factors.. Cell.

[5] Takahashi K, Yamanaka S (2006). Induction of pluripotent stem cells from mouse embryonic and adult fibroblast cultures by defined factors.. Cell.

[6] Loh YH, Agarwal S, Park IH, Urbach A, Huo H (2009). Generation of induced pluripotent stem cells from human blood.. Blood.

[7] Park IH, Arora N, Huo H, Maherali N, Ahfeldt T (2008). Disease-specific induced pluripotent stem cells.. Cell.

[8] Park IH, Zhao R, West JA, Yabuuchi A, Huo H (2008). Reprogramming of human somatic cells to pluripotency with defined factors.. Nature.

[9] Raya A, Rodriguez-Piza I, Guenechea G, Vassena R, Navarro S (2009). Disease-corrected haematopoietic progenitors from Fanconi anaemia induced pluripotent stem cells.. Nature.

[10] Pollock JD, Williams DA, Gifford MA, Li LL, Du X (1995). Mouse model of X-linked chronic granulomatous disease, an inherited defect in phagocyte superoxide production.. Nat Genet.

[11] Alexander BL, Ali RR, Alton EW, Bainbridge JW, Braun S (2007). Progress and prospects: gene therapy clinical trials (part 1).. Gene Ther.

[12] Grez M, Reichenbach J, Schwable J, Seger R, Dinauer MC (2010). Gene Therapy of Chronic Granulomatous Disease: The Engraftment Dilemma.. Mol Ther.

[13] Ott MG, Schmidt M, Schwarzwaelder K, Stein S, Siler U (2006). Correction of X-linked chronic granulomatous disease by gene therapy, augmented by insertional activation of MDS1-EVI1, PRDM16 or SETBP1.. Nat Med.

[14] Stein S, Ott MG, Schultze-Strasser S, Jauch A, Burwinkel B (2010). Genomic instability and myelodysplasia with monosomy 7 consequent to EVI1 activation after gene therapy for chronic granulomatous disease.. Nat Med.

[15] Hotta A, Ellis J (2008). Retroviral vector silencing during iPS cell induction: an epigenetic beacon that signals distinct pluripotent states.. J Cell Biochem.

[16] Okada M, Yoneda Y (2010). The timing of retroviral silencing correlates with the quality of induced pluripotent stem cell lines.. Biochim Biophys Acta.

[17] Schenke-Layland K, Rhodes KE, Angelis E, Butylkova Y, Heydarkhan-Hagvall S (2008). Reprogrammed mouse fibroblasts differentiate into cells of the cardiovascular and hematopoietic lineages.. Stem Cells.

[18] Schenke-Layland K, Angelis E, Rhodes KE, Heydarkhan-Hagvall S, Mikkola HK (2007). Collagen IV induces trophoectoderm differentiation of mouse embryonic stem cells.. Stem Cells.

[19] Yamashita J, Itoh H, Hirashima M, Ogawa M, Nishikawa S (2000). Flk1-positive cells derived from embryonic stem cells serve as vascular progenitors.. Nature.

[20] Nishikawa SI, Nishikawa S, Hirashima M, Matsuyoshi N, Kodama H (1998). Progressive lineage analysis by cell sorting and culture identifies FLK1+VE-cadherin+ cells at a diverging point of endothelial and hemopoietic lineages.. Development.

[21] Nakano T, Kodama H, Honjo T (1994). Generation of lymphohematopoietic cells from embryonic stem cells in culture.. Science.

[22] Vodyanik MA, Bork JA, Thomson JA, Slukvin II (2005). Human embryonic stem cell-derived CD34+ cells: efficient production in the coculture with OP9 stromal cells and analysis of lymphohematopoietic potential.. Blood.

[23] Spangrude GJ, Heimfeld S, Weissman IL (1988). Purification and characterization of mouse hematopoietic stem cells.. Science.

[24] Deng J, Shoemaker R, Xie B, Gore A, LeProust EM (2009). Targeted bisulfite sequencing reveals changes in DNA methylation associated with nuclear reprogramming.. Nat Biotechnol.

[25] Maherali N, Sridharan R, Xie W, Utikal J, Eminli S (2007). Directly reprogrammed fibroblasts show global epigenetic remodeling and widespread tissue contribution.. Cell Stem Cell.

[26] Santilli G, Almarza E, Brendel C, Choi U, Beilin C (2010). Biochemical Correction of X-CGD by a Novel Chimeric Promoter Regulating High Levels of Transgene Expression in Myeloid Cells.. Mol Ther.

[27] Saeki K, Saeki K, Nakahara M, Matsuyama S, Nakamura N (2009). A feeder-free and efficient production of functional neutrophils from human embryonic stem cells.. Stem Cells.

[28] Yokoyama Y, Suzuki T, Sakata-Yanagimoto M, Kumano K, Higashi K (2009). Derivation of functional mature neutrophils from human embryonic stem cells.. Blood.

[29] Morishima T, Watanabe KI, Niwa A, Fujino H, Matsubara H (2010). Neutrophil differentiation from human-induced pluripotent stem cells.. J Cell Physiol.

